# 563 Fire safety in homeless encampments

**DOI:** 10.1093/jbcr/irac012.191

**Published:** 2022-03-23

**Authors:** Sarah Rehou, Greg Cook, Nathan Doucet, Marc G Jeschke

**Affiliations:** Sunnybrook Health Sciences Centre, Toronto, Ontario; Sanctuary Toronto, Toronto, Ontario; ESN, Toronto, Ontario; U of T, Toronto, Ontario

## Abstract

**Introduction:**

It is well known that people experiencing homelessness are at a greater risk for burn injury. Our burn centre saw an increase in admissions of homeless individuals during the pandemic. Typically, we partner with our hospital’s communications staff to share burn prevention public service announcements. But our usual method of broadcasting information through media like newspapers, blog posts, Facebook, or Instagram was not necessarily going to reach people sleeping rough. This report describes the development of a partnership between a burn centre, outreach workers, and people with lived experience of homelessness to improve fire safety in encampments.

**Methods:**

Our goal was to create a Fire Safety Manual and hold Fire Safety Training Sessions. We conducted surveys that asked encampment residents questions like, “What do you use fires for?” “What fire hazards do you see at encampments?” and “How do you think fires could best be prevented?”. We used the results of this survey to guide the training manual and held workshops to engage encampment residents and incorporate feedback into the manual.

**Results:**

The manual uses harm reductions strategies and focuses on real-life situations encountered by folks living outdoors—the manual outlines how to safely start a fire and what to do if a fire occurs. The reality is that people are trying to survive freezing winters while sleeping outside; this means that some safety standards are not possible, and the guide had to reflect that. For example, we practiced fire escape plans during training sessions and had to think about obstacles like tents with only one way out. A solution was to keep a utility knife inside and outside the tent in case one had to cut through to escape or free someone. An encampment resident suggested hiding the knives so they would not be used as weapons. We purchased fire extinguishers, fire blankets, and first aid kits that we distributed during training.

**Conclusions:**

Education is critical to prevent burn injuries. Burn centre staff may be experts on burn prevention, but we are not experts on surviving outside. We have to be accountable to this community. This means listening, building trust, and partnering with people living outdoors. People who did training sessions were empowered to start fire brigades in their encampments. Crucial concepts are to meet people where they are and always to include people with lived experience: “Nothing about us without us.”

